# A novel biomechanical model for reproducing and analysing the causal mechanisms of PFNA “cut-in” phenomenon

**DOI:** 10.1007/s00402-026-06398-x

**Published:** 2026-07-09

**Authors:** Kumaran Rasappan, K. Joshua, Siaw Meng Chou, Leanne Kayla Rebecca Mei-Yi Shaw, Daran Huang, Andy Yew, Ernest Beng Kee Kwek

**Affiliations:** 1 Department of Orthopaedic Surgery, National University Hospital, Singapore, Singapore; 2 Ministry of Health Holdings (MOHH), Singapore, Singapore; 3 School of Mechanical & Aerospace Engineering, Nanyang Technological University, Singapore, Singapore; 4 Division of Musculoskeletal Sciences, Singapore General Hospital, Singapore, Singapore; 5 Department of Orthopaedic Surgery, Woodlands Hospital, Singapore, Singapore

**Keywords:** Intertrochanteric fracture, Cephalomedullary nail, Proximal femoral nail antirotation (PFNA), Bidirectional loading model, Cut-through, Cut-in

## Abstract

**Introduction:**

“Cut-in” is a rare but serious complication associated with cephalomedullary nail fixation for intertrochanteric hip fractures, particularly with Proximal Femoral Nail Antirotation (PFNA). This phenomenon involves paradoxical superomedial migration of the helical blade through the femoral head. Although increasingly recognised, the underlying mechanisms remain incompletely defined. This study aims to describe a new superolateral tensile loading set up in a bidirectional loading model, with the aim of incorporating possible mechanical deviations introduced by abductor weakness.

**Materials and methods:**

This study was conducted in two stages. An initial stage with synthetic femurs (SYNBONE^®^ 2420) was used to calibrate the set up. Within this stage, intramedullary canal diameter was compared (12 mm vs. 18 mm). In the final stage, 5 osteoporotic synthetic femurs (Sawbones^®^ 3503) were used. In both stages, standardised AO/OTA 31-A1.1 intertrochanteric fracture were created in the synthetic femurs and fixed with PFNA-II implants. All femurs were subjected to cyclical bidirectional loading with a universal testing machine. Each cycle consisted of axial compression (720 N) and superolateral tensile loading (120 N) at 2 Hz. This configuration was designed to simulate altered hip biomechanics postulated with the trendelenburg gait. Testing was continued until mechanical failure, construct deformation, or specimen fracture occurred. Full cut-in was defined as complete perforation of the blade through the femoral head cortex, while partial cut-in was defined as superomedial migration of the blade without perforation of the femoral head cortex.

**Results:**

The initial stage demonstrated partial cut-in more frequently in specimens with larger canal diameters (18 mm), but did not produce any full cut-in. The final stage with an 18 mm canal diameter achieved full cut-in in all five specimens.

**Conclusions:**

Our bidirectional loading model with superolateral tensile loading reproduced full ‘cut-in’ in all 5 specimens of the final stage, serving as a proof-of-concept tool. Larger studies are warranted for validation.

## Introduction

Osteoporotic hip fractures remain a major global health concern, with over 10 million cases in 2019 [1] and predictions of this number doubling by 2050 [2]. Associated with significant morbidity and mortality, hip fractures also greatly impact quality of life [3]. Intertrochanteric fractures, arising in the extracapsular region of the femur between the greater and lesser trochanter, account for 50% of all hip fractures [4]. These are associated with a 1-year mortality of 15–20% [5]. 

Various fixation devices have been developed for intertrochanteric fractures to improve fracture union rates, facilitate early weight-bearing and in so doing, reduce morbidity and mortality from prolonged bed rest [6, 7]. Historically, the DePuy Synthes Dynamic Hip Screw (DHS) was widely used for intertrochanteric fracture fixation due to its effectiveness in stabilizing simple fractures [8]. However, in recent years, the DePuy Synthes Proximal Femoral Nail Antirotation (PFNA) has gained popularity, thanks to reduced blood loss from its minimally invasive approach, shorter operative time as well as fewer complications [9]. The PFNA also achieves better angular and rotational stability due to its intramedullary design, especially for multifragmented unstable fractures (AO 31A2-3) [10–12]. 

Biomechanically, the PFNA features a single helical blade with a sharp tapered tip designed to compact cancellous bone during malleted insertion, ensuring excellent fit within the femoral head while minimizing bone removal compared to traditional screws [11, 13], which require more reaming. Postoperative repetitive weight-bearing on the femur promotes further dynamic compression at the fracture site [14]. The structure of the PFNA blade allows for dynamic compression with rotational stability [11, 15], while DHS requires an additional antirotation screw to achieve similar rotational stability [16]. The intramedullary PFNA is also closer to mechanical axis of the femur, resulting in more effective load-sharing with the native bone, reduced bending forces and improved overall biomechanical stability when compared with the extramedullary DHS during dynamic compression. However, the PFNA is associated with increased risk of implant complications, especially if the implant is poorly placed [17]. 

Cut-out, a common implant-related complication in hip fractures, involves perforation of the femoral head or neck superiorly by the lag screw or blade, due to varus collapse at the neck shaft junction [18]. Previous studies introduced Tip–Apex Distance (TAD), a measurement between the lag screw tip and the femoral head apex [19], and described an increased cut-out risk with a measurement over 25 mm in DHS [19–22]. The position of the blade in the femoral head, also known as Cleveland zones, was also described as a predictor of cut-out [23]. While less established for PFNA, Yam et al. proposed an adapted TAD for the helical blade, suggesting it may also predict cut-out risk in intramedullary fixation [24]. 

A complication that has become increasingly reported with cephalomedullary nail devices such as the PFNA is the ‘cut-in’, or ‘cut-through’ phenomenon. A literature review by Law et al. revealed that there were 16 such clinical cases in a period of 10 years from 2006 to 2017, of which 5 were seen in PFNA [25]. This complication refers to the paradoxical tendency for the blade to slide superomedially, as opposed to the intended inferolateral movement of the blade which allows controlled collapse at fracture site during weight bearing. Sequelae of this mechanism of failure include femoral head perforation, acetabular perforation and migration of implant into the pelvic cavity [26]. 

One postulation for the reason why this occurs in PFNA and not in DHS is the sharper tapered blade tip which is malleted in compared to the screw that is primarily screwed in. Furthermore, the DHS plate is fixed onto the lateral cortex, limiting ratcheting of the screw. The PFNA on the other hand, is intramedullary, and prevents movement only in the presence of an intact lateral wall. It must be noted that there exists a scarcity of current research into the mechanism behind the ‘cut-in’ phenomenon. Previous attempts at reproduction of ‘cut-in’ are seen with Weil et al., [27] with unidirectional loading. Another study by Law et al. [25] pioneered bidirectional loading with the introduction of the tensile loading. Both reproduced the phenomenon by measuring the medial migration distance of the PFNA blade within the femoral head. These two studies have postulated mechanisms by which the failure occurs within their parameters. However, complete cut-in with perforation of the femoral head is yet to be achieved consistently in biomechanical studies. Furthermore, these studies were performed with the assumption that the post-surgical hip behaves like a normal hip, with no abductor weakness that might result in Trendelenburg gait.

Our study aims to describe a new superolateral tensile loading set up in a bidirectional loading model, with the aims of incorporating possible mechanical deviations introduced by abductor weakness.

## Methods

This study was conducted in two stages, initial calibration to test the robustness of the set up and optimise parameters, followed by a focused investigation to consistently reproduce full cut-in under controlled conditions.

### Specimens and fracture simulation

The initial calibration stage employed synthetic femurs (Model 2420, SYNBONE^®^, SYNBONE AG, Zizers, Switzerland) composed of a hard polyurethane-based core and polyurethane-based rigid composite cortex. For the final stage, 5 synthetic femurs better representing osteoporotic bone mechanics (Model 3503, Sawbones^®^, Pacific Research Laboratories, Vashon Island, WA, USA), composed of low density solid rigid polyurethane foam core with a short fiber filled epoxy cortex, were used. Standardized isolated intertrochanteric fractures (AO/OTA type 31-A1.1) [28] were created in all specimens using a precision 1.27 mm thin band saw.

In the final stage, a 60 ± 7 Shore-A elastomer with 3 mm thickness was inserted between fracture fragments to simulate soft tissue tension as well as to replace the bone loss from the saw and replicate nail-bone elasticity, as previously described by Weil et al. [27] and Law et al. [25]

### Implant Fixation

All specimens were reduced and fixed using the standard DePuy Synthes PFNA-II surgical technique. A short PFNA implant (Ti-6Al-7Nb alloy) comprising a 9 mm diameter with 200 mm length nail, 100 mm length blade, and 5 mm diameter with 30 mm length distal locking screw was used. To ensure identical nail blade position and nail rotation in both the AP and axial planes, a guidewire was inserted in a retrograde fashion from the manufacturer-designed central hole in the femoral head past the lateral cortex through the PFNA jig to ensure uniformity of insertion in all models. Thereafter, a blade was inserted via the guidewire through standard techniques. In addition, we used consistent landmarks on the specimen and standardised jigs which allowed for standardised positioning across all specimens while ensuring that the tip of the blade was 1 cm away from the femoral cortex. The distal screw was placed in static locking mode to increase consistency across all specimens.

### Mounting and alignment

**Fig. 1:**
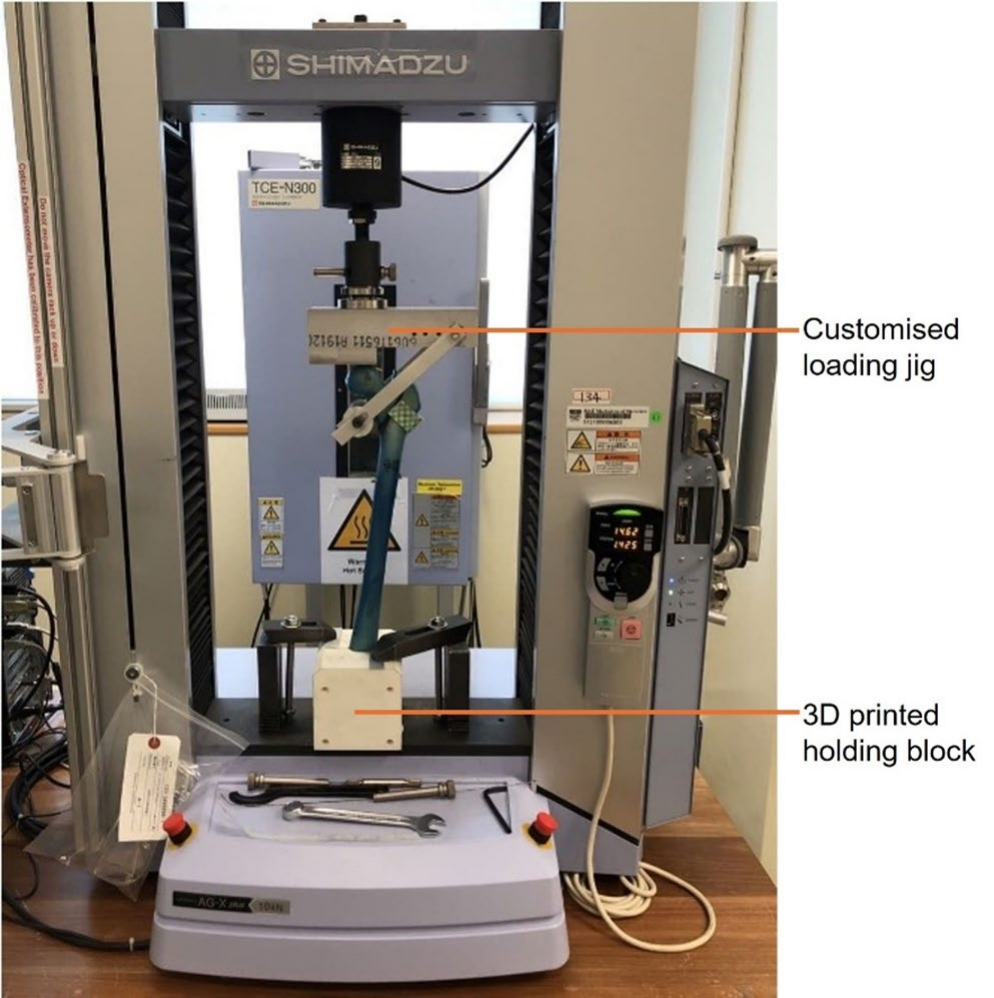
Final stage experimental set up with Sawbones^®^ Product no. 3503

To ensure consistent biomechanical positioning, one distal femur each from the SYNBONE^®^ 2420 models and the Sawbones^®^ 3503 models were scanned using a three-dimensional (3D) scanner (Artec Leo, Artec 3D, Luxembourg) and reverse 3D scanning was done. For both stages, 3D-printed holding blocks were created based on scan data. In the final stage, a custom bipartite base fixture was developed to fit the femoral condyles to further reduce rotational instability, and this was set at a physiological valgus alignment of 6 degrees (Fig. 1) [29]. 

### Biomechanical testing

Mechanical testing was performed using an universal testing machine (Shimadzu^®^ Autograph AG-X 10 kN, Kyoto, Japan) and number of cycles to failure was recorded using machine’s operating software (Shimadzu^®^ TRAPEZIUMX v1.5.1, Kyoto, Japan). A cyclical axial compressive load of 720 N was applied to the femoral head using a load applicator, simulating the body weight of a 70 kg individual during the stance phase. Between each compressive axial load, a tensile load of 120 N was applied at the inferomedial aspect of the femoral head, delivered in a superolateral direction at an angle of 45 degrees from the horizontal plane via a custom load applicator (Fig. 1). This approximated hip traction forces from the weight of a single lower limb (1/6th body weight) [30] during the swing phase of a patient with Trendelenburg gait. This angle was chosen empirically as a proof-of-concept parameter, to reproduce a hypothesised superolateral load that occurs during Trendelenburg gait. A frequency of 2 Hz with sinusoidal waveform was used.

Cyclical testing was continued until mechanical failure occured. This was defined as having any one of the following:

- Visible construct deformation.
- Superomedial blade perforation through the femoral head apex (full cut-in).
- Superior or superolateral blade perforation through femoral head or neck (full cut-out).
- Specimen failure (defined as any fractures within the specimen).

For constructs which failed without having full cut-in, the specimen was bisected. The distance from the blade tip to the femoral cortex was measured with a ruler, and if it was less than 1 cm, partial cut-in was determined to have occurred. Following failure, all femoral heads were coronally bisected and inspected for cut-in, cut-out, or both.

### Variables assessed

During the initial calibration stage, attempts were made to test variables previously identified in the literature as contributing to PFNA implant failure [20, 31–35] to evaluate their potential influence on cut-in. These included neck shaft angle, nail entry point, tip apex distance and intramedullary canal diameter.

However, during initial calibration, several specimens failed prematurely due to excessive loading, limiting the amount of usable data obtained. The only variable that generated usable data was intramedullary canal diameter (12 mm vs. 18 mm), evaluated across 10 specimens. 

## Results

Full cut-in was not observed in any of the specimens used in the initial calibration stage. These specimens were noted to have failed through fractures prior to observation of full cut-in. Bisection of these specimens was performed to observe for partial cut-in.

The 18 mm canal diameter group exhibited partial cut-in in 4 out of the 5 specimens tested, while the 12 mm diameter group exhibited cut-in in only 2 out of the 5 specimens tested. Both groups had 1 immediate failure each upon loading through specimen fracture (Table 1). These results indicate a potential association between the occurrence of cut-in and wider canal diameter. However, this trend was not statistically evaluated due to the limited sample size, as this exploratory stage was intended to optimise the experimental parameters for the final stage of the study. As such, no mechanistic conclusion can be derived from this data.

**Table 1:** Pilot stage results comparing Intramedullary canal diameter

Specimen No. (1–10)	Intramedullary Canal Diameter (mm)	Presence of partial cut-in	Number of cycles to failure
1	12	No	–
2		*Yes*	2840
3		No	2085
4		*Yes*	2557
5		No	2513
6	18	*Yes*	2651
7		*Yes*	2985
8		*Yes*	2138
9		*Yes*	1800
10		No	–

Immediate failure

**Fig. 2:**
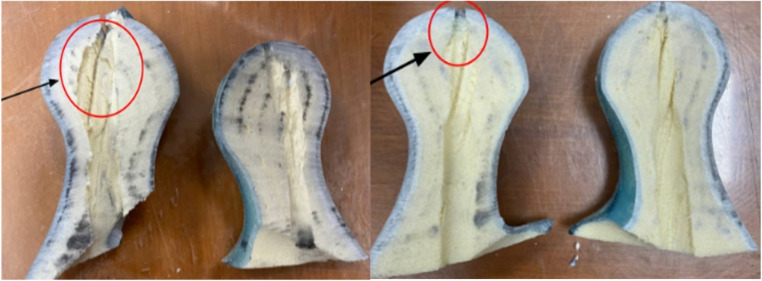
Cross section Synbone^®^ Product no. 3503 used in final phase with full cut-in observed

Subsequent testing with Sawbones^®^ 3503 models with an 18 mm canal diameter resulted in full cut-in as the only failure mode in all 5 of the constructs (Fig. 2). The mean number of cycles to failure was 2256 (Table 2). Although all five specimens demonstrated full cut-in, the limited sample size resulted in a wide 95% confidence interval (5/5, 100%; 95% CI 47.8%–100%).

**Table 2:** Final stage results

Specimen No. (1–5)	Intramedullary Canal Diameter (mm)	Presence of full cut-in	Number of cycles to failure
1	18	*Yes*	1962
2		*Yes*	2218
3		*Yes*	2113
4		*Yes*	2984
5		*Yes*	2005
**-**		Average	2256

## Discussion

Biomechanical testing protocols for the reproduction of the cut-in phenomenon remain scarce in the current literature. Weil et al.’s initial radiographic analysis of eight PFNA fixations with cut-in revealed a common trend: a compromise in the greater trochanter and the medial calcar buttress resulting in a wider proximal femoral canal [27]. During the stance phase, axial loading is transmitted via the medial aspect of the helical blade. When the resistance of the superomedial trabecular bone is inadequate relative to the applied load, medial migration of the blade, i.e. cut-in, may occur. During swing phase, when the compressive load is reduced, the abovementioned wider proximal femoral canal resulted in the nail falling back laterally while friction between the helical blade and the surrounding bone was proposed to hold the helical blade in place. This repetitive toggling mechanism with each gait cycle was proposed to be the cause of cut-in, and Weil et al. reproduced cut-in with repetitive axial loading with an allowed increased movement in the proximal nail in five different intramedullary devices (Proximal Femoral Nail, Proximal Femoral Nail Antirotation, Trochanteric Fixation Nail, Intramedullary Hip Screw, Gamma 3 Trochanteric Nail System). Proximal nail movement restriction resulted in obliteration of cut-in in four of the implants with a single helical blade, only being observed in the PFN which featured two helical blades.

**Fig. 3:**
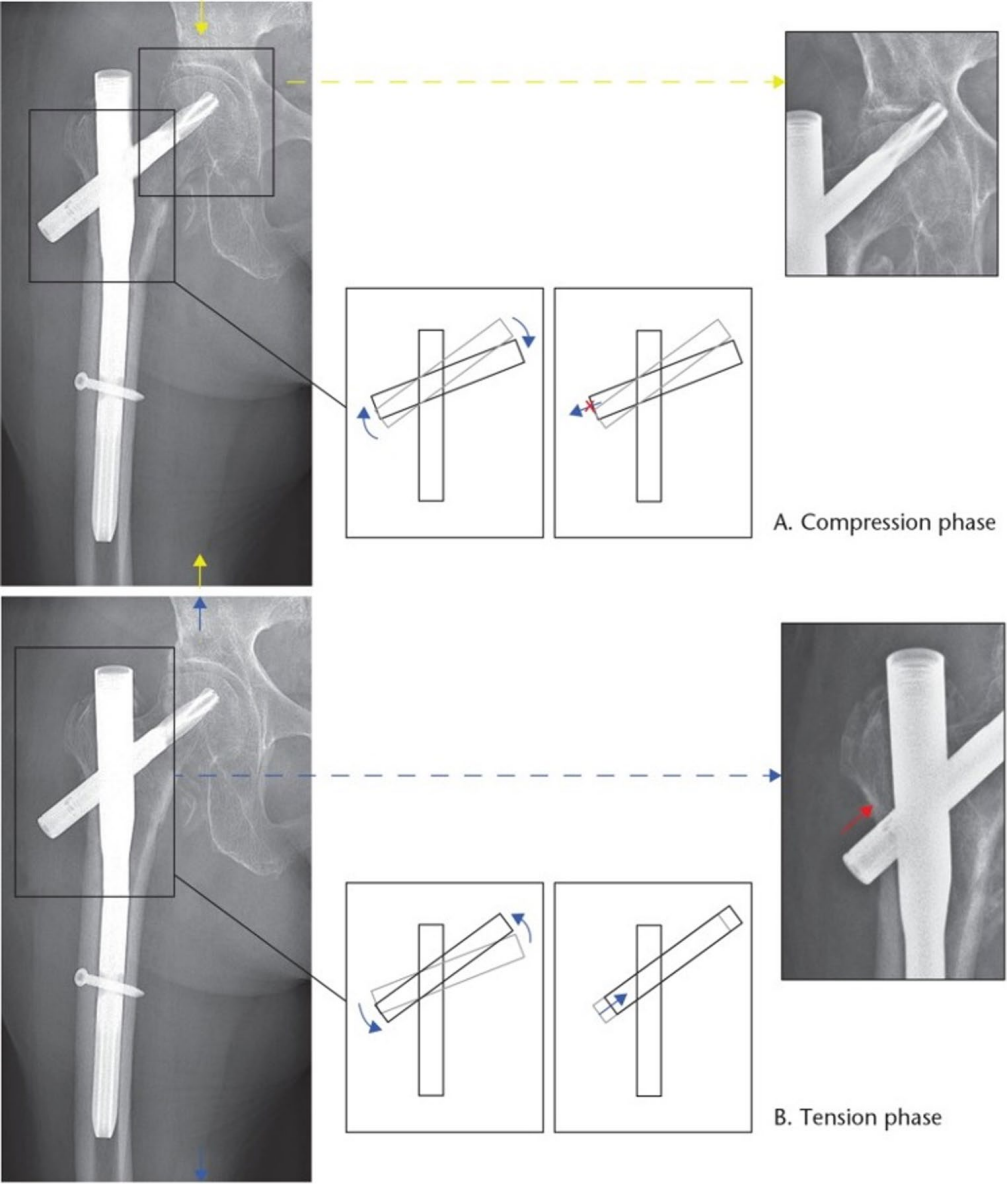
Diagrammatic representation of postulated mechanism behind the cut-in phenomenon with toggling mechanism. Adapted from “Medial migration in cephalomedullary nail fixation of pertrochanteric hip fractures,” by Law. GW, Wong. YR, Yew. AK, Choh. ACT, Koh. JSB, Howe. TS, 2014, Bone Joint Res. 2019 Aug 2;8(7):313–322. (10.1302/2046-3758.87.BJR-2018-0271.R1). Copyright 2019 by authors et al.

Law et al. [25] proposed the following hypothesis to explain the role of tensile loading. During the stance phase, the blade was postulated to cut-in through the femoral head as mentioned above. However, the blade was also postulated to generate a moment in the varus direction of the construct, essentially “locking” the construct in place and preventing lateral migration of the nail with respect to the blade. During swing phase, the blade was hypothesised to generate a valgus moment as the load was reduced and tensile loading was applied, unlocking the construct and allowing for superomedial propagation of the blade with respect to the nail. They proved this hypothesis with their bidirectional loading model which was seen to have a significantly higher rate of cut-in with an increased medial migration distance compared to their unidirectional loading model. The provided figure, courtesy of Law et al., demonstrates this concept. [25] (Fig. 3).

Our biomechanical testing protocol for the final stage in our study was built upon the foundation laid by these two authors, with a modified approach to better represent potential biomechanical differences which may occur in a post-surgical hip.

Abductor muscle weakness, specifically gluteus medius weakness, is a well-documented phenomenon post-surgical fixation of proximal femur fractures with intramedullary nailing [36–40]. CT (computed tomography) evaluation of the gluteus medius revealed significant reduction in its cross-sectional area post-PFNA fixation [37] which translates to weaker hip abduction [39]. 

The Trendelenburg gait is characterised by an adducted hip position on the affected side during stance phase and a characteristic contralateral pelvic drop with a compensatory trunk lean [41–43]. During stance phase of the unaffected limb, the pelvis returns to a level position due to intact abductor function, and the trunk lean is no longer observed. As the affected limb transitions from stance to swing, we postulate that persistent abductor weakness in the hip results in the limb remaining in a relatively adducted position. This is observed clinically with the foot of the affected limb falling closer to the midline of the body when there is reduced gluteus medius activation [44–45]. While direct biomechanical evidence specifically examining the affected limb with Trendelenburg gait during swing phase is limited, our hypothesisaligns with established principles of gait biomechanics and compensatory postural strategies.

**Fig. 4:**
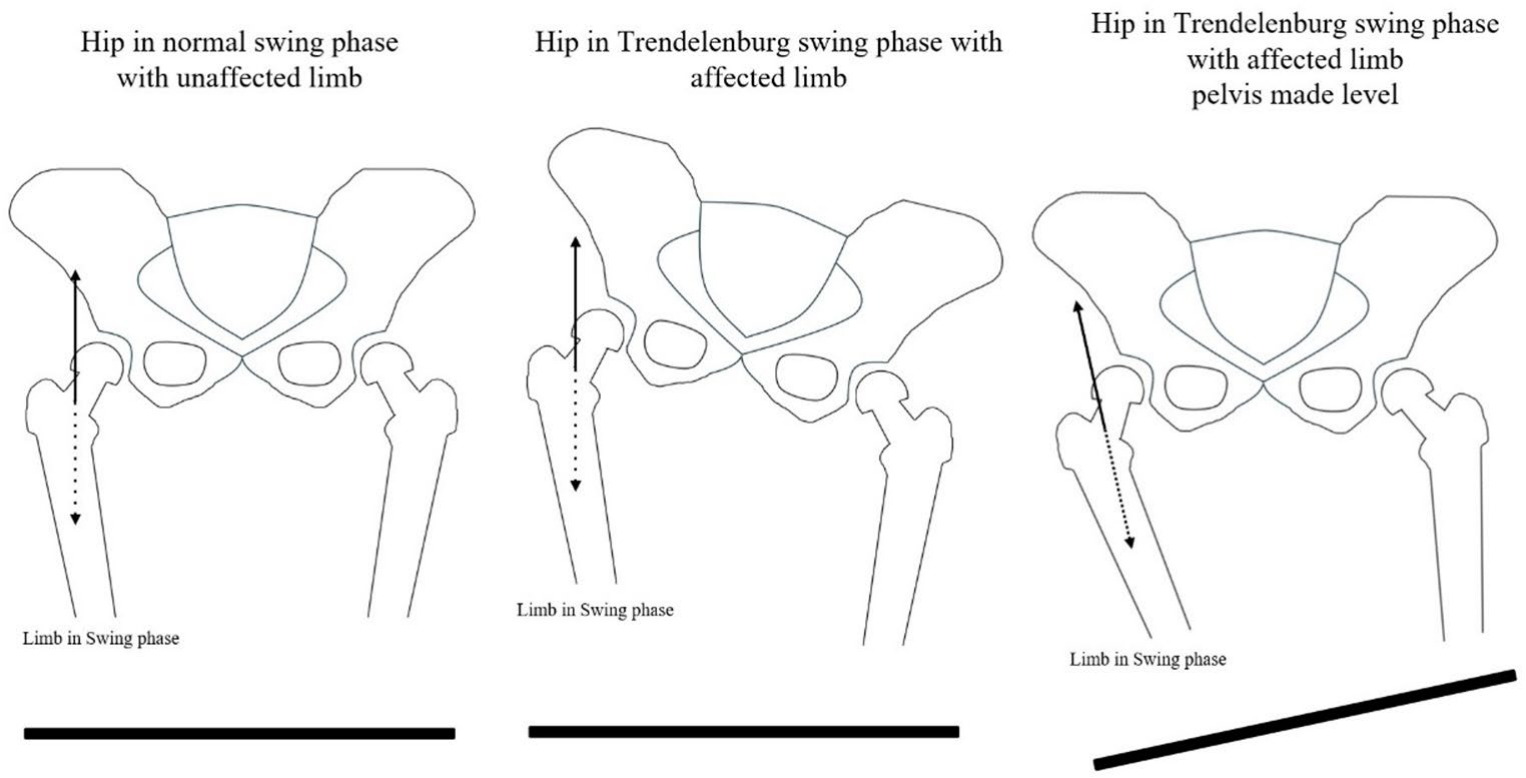
Diagrammatic representation of postulated superolateral tensile load during swing phase in Trendelenburg gait. Dotted arrow represents gravitational force acting on limb; Solid arrow represents resultant tensile load. Solid line represents level ground for reference

Our hypothesis is that the resultant tensile forces acting on the hip joint would be directed superolaterally rather than purely superiorly as seen in the normal hip [46–48]. In the coronal plane, when the pelvis is level, gravity and momentum act inferiorly, generating a superior-directed tensile load due to the limb’s weight and pelvis position. However, in the Trendelenburg gait, when the hip remains in the adducted position during swing phase, we postulate that the gravity and momentum act in an inferomedial direction with relation to the hip joint, resulting in an opposing superolateral tensile load (Fig. 4).

**Fig. 5:**
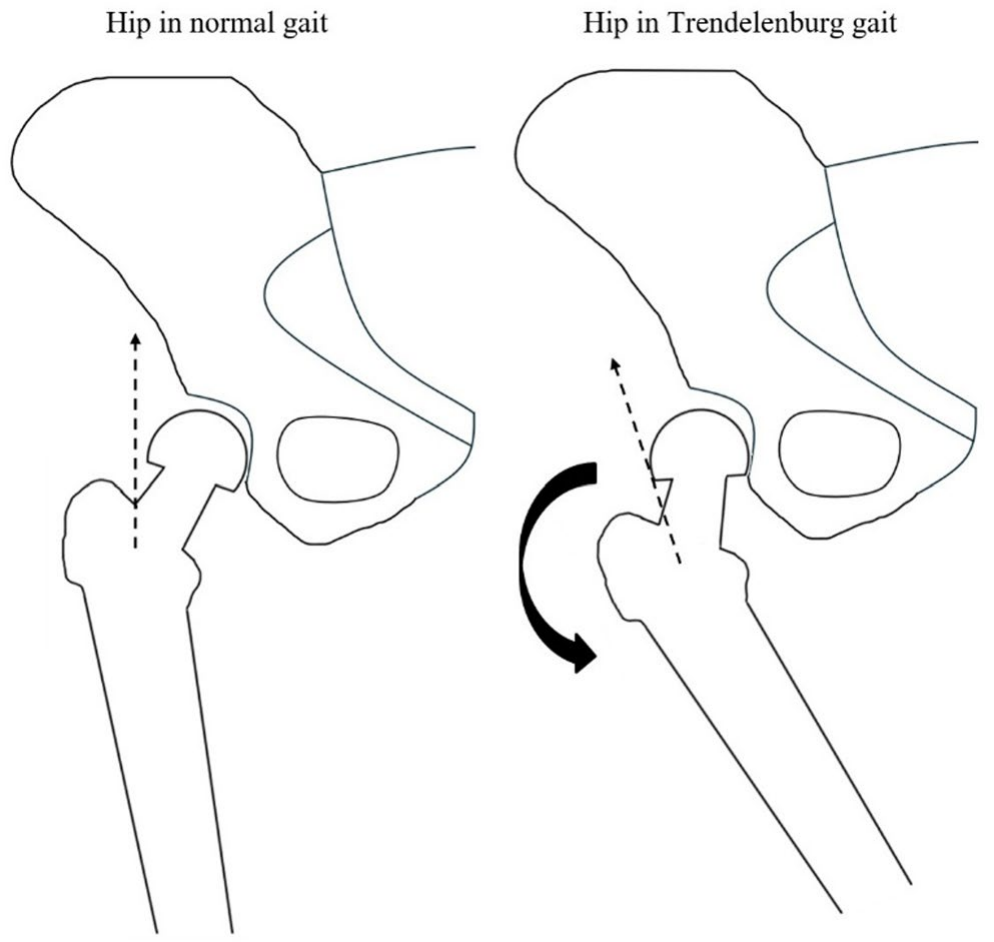
Diagrammatic representation of postulated altered joint interaction within the hip during swing phase in trendelenburg gait. Solid arrow represents postulated movement of femur created by altered joint interaction and gravity; Dotted arrow represents postulated resultant tensile load with further stretch in lateral muscles

Furthermore, we propose a theory that the Trendelenburg gait alters femoral head positioning such that the inferomedial aspect of the femoral head may encounter, and lever against the inferior margin of the acetabular rim during swing phase due to the adducted nature of the hip. This altered joint interaction could further propagate a moment arm in the adduction direction of the hip with gravity being the driving force. This translates to further stretching of the abductors and lateral soft tissues of the hip, resulting in greater lateralisation of the tensile force (Fig. 5). Further biomechanical studies are required to explore and validate this theory.

Our study included a superolateral tensile load originating from below the inferomedial aspect of the femoral head, to simulate both the moment arm as described above from the altered joint interaction as well as the superolateral tensile load due to gravity acting on the limb. An angle of 45 degrees from the horizontal plane was used for this superolateral tensile load, to account for the adducted nature of the hip as well as the altered joint interaction to reflect the pivoting of the femoral head over the inferior edge of the acetabulum. The angle chosen was a proof-of-concept value to reflect the compounded effect of hip adduction and pelvic tilt, to achieve a higher full cut-in rate. Precise in-vivo measurement or Finite Element Analysis modelling is recommended in further studies for further calibration of this angle.

**Fig. 6:**
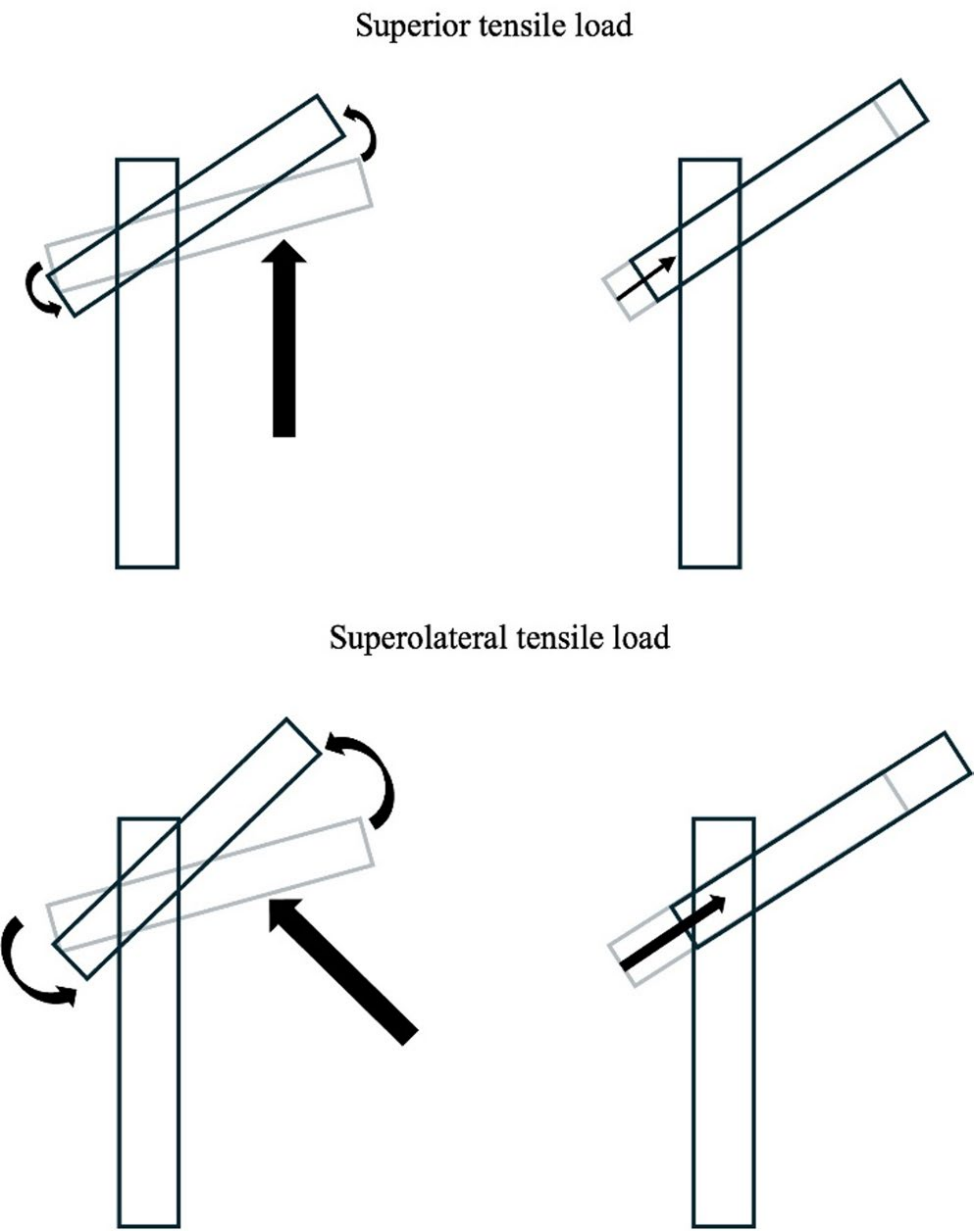
Diagrammatic representation of postulated increased valgus force from superolateral tensile load resulting in increased superomedial propulsion of PFNA helical blade

A superolateral tensile load can amplify the resultant valgus moment on the blade during swing phase. We hypothesise that this allows for a greater superomedial propagation of the blade before the next compressive loading. (Fig. 6). Increased superomedial propagation of the blade then potentially allows for further cut-in during compressive loading, a similar mechanism to that described in Weil et al. With this simple modification to the set-up, we produced full cut-in of the blade through the femoral head in all 5 synthetic femurs in our final stage. Despite the wide confidence interval, these findings nonetheless establish consistent reproduction of full cut-in within this proof-of-concept series. However, larger scale studies will be required to validate these results.

An experimental setup most comparable with our own is the bidirectional loading model described by Law et al. Key similarities include the tensile load (120 N), the use of elastomer with similar properties (70 A vs. 60 ± 7 A), overreaming of the bone canal (18 mm in our study vs. unspecified in theirs). Notable differences include the compressive load (600 N vs. 720 N), synthetic femur density (17 pounds per cubic foot (PCF) vs. 10 PCF), cortical thickness (standard vs. thinned), fracture morphology (complex vs. simple) and direction of tensile load (superior vs. superolateral) [25]. 

Using their setup, Law et al. did not achieve complete cut-in in any of their 6 specimens after 5000 cycles, while our setup reproducibly achieved complete cut-in in all 5 specimens with substantially fewer cycles. Direct statistical comparison between these two setups is not appropriate given the differences in loading parameters, synthetic bone characteristics and construct configurations. The higher density synthetic femurs with thicker cortices likely conferred greater structural resistance, while the lower compressive load may have permitted longer cycling before failure. However, the comminuted fractures theoretically reduced structural resistance and may have had opposite effects to the higher density, thicker cortex and lower compressive load. Therefore, the effect of tensile load direction on cut-in progression remains unclear and warrants further investigation. While an included internal control group would have strengthened causal inference, this was not undertaken due to the exploratory nature of the study and associated resource constraints.

**Fig. 7:**
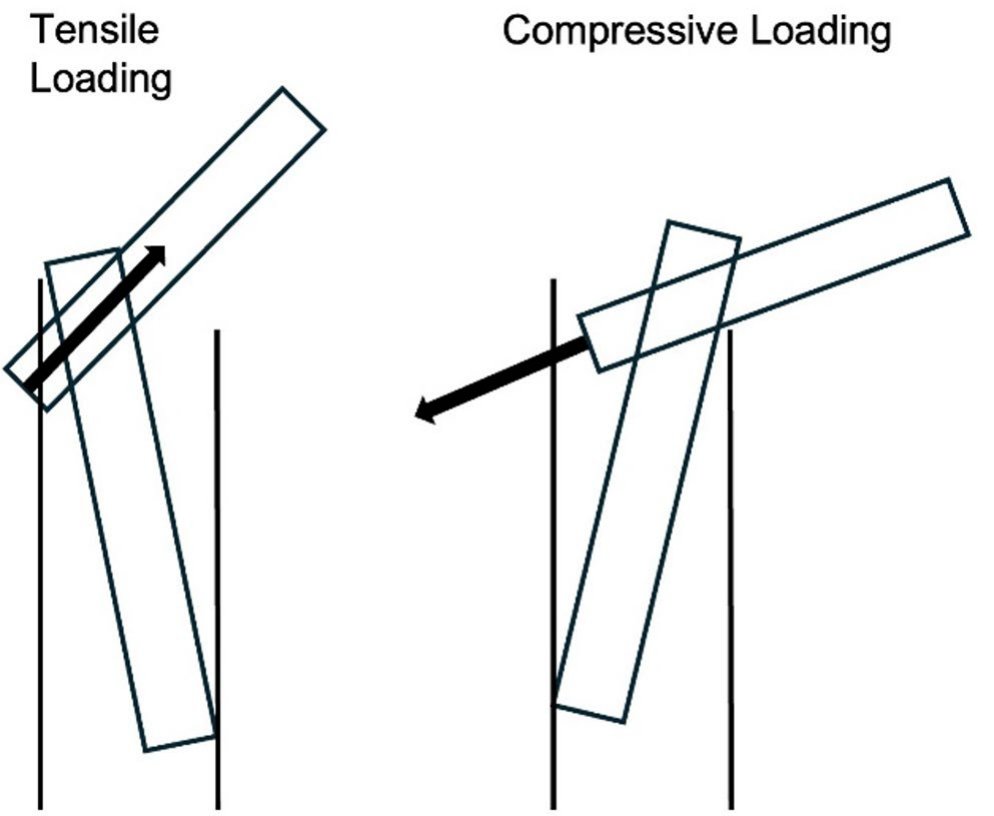
Diagrammatic representation of postulated implant movement within a larger diameter femoral canal and associated blade position. Solid arrow represents direction of movement of blade in each loading phase

The initial stage of this study was conducted to calibrate our setup and attempt to preliminarily assess several variables hypothesized to influence the rate of full cut-in. As mentioned previously, only the intramedullary canal diameter groups were able to generate data which was not statistically evaluated. Our limited data showed a potential association between larger femoral canal diameter and rate of partial cut-in. During tensile loading, we postulate that free movement of the entire implant allows the blade to assume a more vertical position, which allows it to move more freely within its aperture in the nail and hence, achieve greater superomedial propagation. Conversely, during compressive loading, the blade is more perpendicular, and movement within its aperture in the nail is reduced, preventing inferolateral movement (Fig. 7). This additional mechanism could also contribute to the full cut-in seen in all the femurs used in our final stage. Further studies with larger sample sizes will have to be conducted to confirm these findings.

An additional distinction of our study is the use of a simple 31A1.1 type fracture, with an intact greater trochanter and a preserved medial calcar buttress. We were able to replicate full cut-in in all the synthetic femurs with the simplest fracture morphology. Our observation suggests that cut-in may occur even within stable intertrochanteric fractures, potentially in the presence of significant postoperative Trendelenburg gait. Cut-in in stable fracture configurations has only been reported in one series [26]. In contrast, cut-in is observed primarily in unstable intertrochanteric fractures in other preceding literature [26, 49–52]. To our knowledge, this represents one of the first studies to propose such a biomechanical association. Further investigation is required to clarify this relationship.

### Limitations

The sample sizes for our paper were limited, due to a paucity of resources. The utilised SYNBONE^®^ model 2420 femurs in our pilot study were fragile, and had many specimen failures prior to full cut-in. This reduced the data that we could generate with our calibration stage, resulting in prevention of proper statistical analysis. More Sawbones^®^ 3503 models should be used for testing with adequate numbers, to achieve adequate statistical power and to confirm our preliminary finding that increasing canal diameters produce the most cut-ins.

The Sawbones^®^ 3503 models, while designed to replicate the osteoporotic human femur, might be inferior to cadaveric femurs with regards to structural stability and biomechanical properties. However, this bone model was chosen to allow as much uniformity in quality within the models to eliminate this confounder as a factor affecting results regardless of the number of bones being tested.

Our study only approaches the gait cycle from a coronal plane, and there might be effects from the sagittal plane during the swing phase that are not included in the study. These include the effects of the anteriorly located hip flexors (i.e. iliopsoas muscle) and the posteriorly located hip extensors (i.e. gluteus maximus muscles), which are a major group of muscles involved in the swing phase, and are likely to shift the tensile load experienced by the hip joint more anteriorly and posteriorly respectively.

Although current literature reports that hip joint forces can reach 2 to 3 times body weight during this phase, a value of 720 N was selected for compressive load due to limitations in the loading mechanism [53]. Future studies with higher loads might accelerate failure or alter its mode.

Another important limitation to consider is the scarcity of biomechanical studies specifically examining forces experienced within the joint in Trendelenburg gait. Due to limitations in terms of studies which specifically investigate the joint interactions within a hip joint in Trendelenburg gait, especially in swing phase, no exact calculation of the tensile load angle could be made for this study. However, this also becomes a strength of our study as this is one of the first studies available that investigates this phenomenon and attempts to explain this.

The angle and magnitude of the tensile load in this study was empirically chosen as a proof-of-concept value to demonstrate feasibility of our bidirectional loading model. The exact influence of the angle and magnitude cannot be elicited by this study. Further investigations into the angle and magnitude of the proposed superolateral tensile load would allow for determination of the influence that tensile load direction and magnitude has on cut-in rates. Additionally, Finite Element Modelling analysis may also provide valuable insights and allow for modifications that might more accurately portray the precise joint mechanics.

Cement augmentation, which is commonly used in osteoporotic proximal femur fracture management, was also not considered here, as the primary goal was to develop a method that reliably reproduces full cut-ins . This can be considered for further exploration.

## Conclusion

A 45° superolaterally directed hip tensile loading and canal-to-nail diameter mismatch may contribute to PFNA cut-in rates, even within simple fracture morphologies. Our bidirectional loading model with an incorporated superolateral tensile load consistently reproduced complete cut-in within this proof-of-concept series. Larger studies and robust testing of potential contributing variables offer promising insights for future clinical applications and recommendations.

## Data Availability

No datasets were generated or analysed during the current study.
